# Three new species of *Melloleitaoina* Gerschman & Schiapelli, 1960 (Araneae, Mygalomorphae, Theraphosidae) from northern Argentina

**DOI:** 10.3897/zookeys.404.6243

**Published:** 2014-04-24

**Authors:** Carlos Perafán, Fernando Pérez-Miles

**Affiliations:** 1 Universidad de La República, Facultad de Ciencias, Sección Entomología, Iguá 4225, Montevideo, Uruguay

**Keywords:** Tarantula, taxonomy, Theraphosinae

## Abstract

Three new species of the monotypic genus *Melloleitaoina* Gerschman & Schiapelli, 1960 are described from northern Argentina: *M. mutquina* **sp. n.**, *M. uru* **sp. n.** and *M. yupanqui* **sp. n.** The female specimen originally described as *M. crassifemur* is not conspecific with the male holotype and thus is removed from this species and described as *M. uru* **sp. n.**; *M. crassifemur* is redescribed. All species are diagnosed, illustrated and a key to species is provided.

## Introduction

The subfamily Theraphosinae (Theraphosidae) is a speciose group of tarantulas distributed exclusively in the New World, whose greatest diversity is found in South America. Most tarantulas have cryptic habits, are predominantly nocturnal sit-and-wait hunters and have long lifespans. Females can live between 15 and 30 years while males live for significantly shorter periods of time (Locht et al. 1999, Costa and Pérez-Miles 2002). Juveniles and adult females are sedentary while mature males disperse in search of females. Consequently several species have only been described on the basis of a single sex and the subsequent assignment of a specimen as a conspecific of the opposite sex is problematic.

The monotypic genus *Melloleitaoina* was established by Gerschman and Schiapelli (1960) on the basis of a single male specimen of the type species *Melloleitaoina crassifemur*, from Salta, Argentina. Later in 1973 the same authors illustrated the female spermathecae of a specimen from a location near the type locality in the same province. Raven (1985) considered *Melloleitaoina* a junior synonym of *Dryptopelma* Simon, 1889, but was restored by Pérez-Miles et al. (1996). This genus is morphologically similar and phylogenetically related to *Tmesiphantes*, *Plesiopelma* and *Homoeomma* (Pérez-Miles et al. 1996, Yamamoto et al. 2007).

*Melloleitaoina* is characterized by having a reduced number of labial cuspules, legs with few spines, all tarsal scopulae divided and lacking scopulae on metatarsal IV. Males have a thickened femur III, palpal organ with a long and curved embolus, and tibial apophysis with two very unequal branches. Females have spermathecae with two granulated seminal receptacles with a slight constriction near the apex (Gerschman and Schiapelli 1960, Gerschman and Schiapelli 1973, Pérez-Miles et al. 1996, Yamamoto et al. 2007).

Our study of the types and additional material deposited in the Museo Argentino de Ciencias Naturales “Bernardino Rivadavia”, led us to determine that the female of *Melloleitaoina crassifemur* is not conspecific with male holotype when contrasted with males from the same locality of the female. Also we found other individuals that fit with the diagnosis of the genus but are undescribed; they are described as new species herein.

## Material and methods

Urticating setae terminology follows Cooke et al. (1972) and Bertani (2002). Male palpal organ keel terminology follows Bertani (2000). All measurements were taken using an ocular micrometer and are given in millimeters (mm). We measured left legs and palps unless they were lost; measurements were taken in dorsal view along the central axis of the segments. Photographs were taken with a Lumenera Infinity Lite camera adapted to a stereoscopic microscope Olympus SZ 61. The geographic coordinates were determined using the Global Gazetter (www.fallingrain.com). The distribution map was produced using DIVA-GIS 7.5 (www.diva-gis.org). The material examined is deposited in the Museo Argentino de Ciencias Naturales “Bernardino Rivadavia” (MACN).

The following abbreviations are used: ALE = anterior lateral eyes; AME = anterior median eyes; OQ = ocular quadrangle (including lateral eyes); p = prolateral; PB = prolateral branch of tibial apophysis; PI = prolateral inferior keel; PME = posterior median eyes; PMS = posteromedial spinnerets; PLE = posterior lateral eyes; PLS = posterolateral spinnerets; PS = prolateral superior keel; r = retrolateral; RB = retrolateral branch of tibial apophysis.

## Taxonomy

### Genus Melloleitaoina

Gerschman & Schiapelli, 1960

http://species-id.net/wiki/Melloleitaoina

#### Type species.

*Melloleitaoina crassifemur* Gerschman & Schiapelli, 1960

#### Diagnosis.

Both sexes have a reduced number of labial cuspules (6–14), all tarsal scopulae divided and metatarsal IV scopulae absent. Males differ from other Theraphosinae by having a thickened femur III, palpal organ with a long and curved embolus with two prolateral keels (PI and PS) (Figs 3–4, 9–10, 16–17 and 26–27), and tibial apophysis with two very unequal branches (Figs 6, 11, 21 and 29). Females differ from other Theraphosinae by having spermathecae with two granulated seminal receptacles with a slight constriction near the apex (Figs 15 and 25) and spiniform setae on promargin of coxae III and IV (Figs 19 and 20). Females have type IV urticating setae while males have III-IV intermediated urticating setae.

#### Affinities.

*Melloleitaoina* species share with *Plesiopelma* Pocock, 1901 and *Tmesiphantes* Simon, 1892, principally by the general morphology of the palpal bulb and tibial apophysis. *Melloleitaoina* males can be distinguished additionally from those of *Plesiopelma* by lacking nodule on metatarsi I and having only III-IV intermediated urticating setae. They can be distinguished from *Tmesiphantes* by having sigillas more rounded, male femur III incrassate and female spermathecae with granulated seminal receptacles and spiniform setae on promargin of coxae III and IV.

#### Distribution.

**Figure 1.**
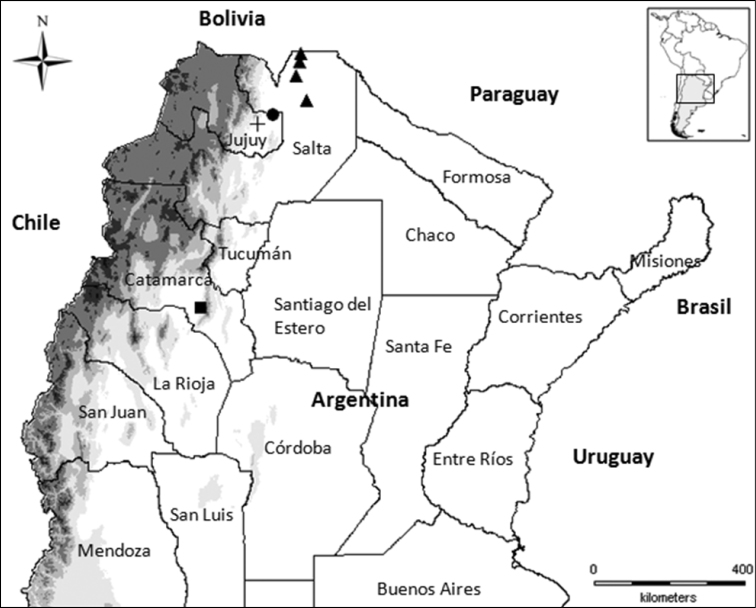
Map of Northern Argentina, geographical distribution of the *Melloleitaoina* species. *Melloleitaoina uru* (triangles); *Melloleitaoina crassifemur* (circle); *Melloleitaoina yupanqui* (cross); *Melloleitaoina mutquina* (square).

Northern Argentina. Catamarca, Salta and Jujuy provinces (Fig. 1).

### Melloleitaoina crassifemur

Gerschman & Schiapelli, 1960

http://species-id.net/wiki/Melloleitaoina_crassifemur

Figs 2–6

#### Material examined.

Only type material.

#### Type material.

Holotype male from Argentina, Salta, Orán, Urundel, 335m above sea level, 23°33'0"S, 64°25'0"W, viii-1947, Misión Ricardo N. Orfila leg. (MACN-Ar 2285).

#### Diagnosis.

Male differs from other *Melloleitaoina* species by the palpal bulb morphology with very curved embolus without triangular tooth, well-developed and subequal PI and PS (Figs 3 and 4), and apex widened (Fig. 5). Females unknown.

**Figures 2–6.**
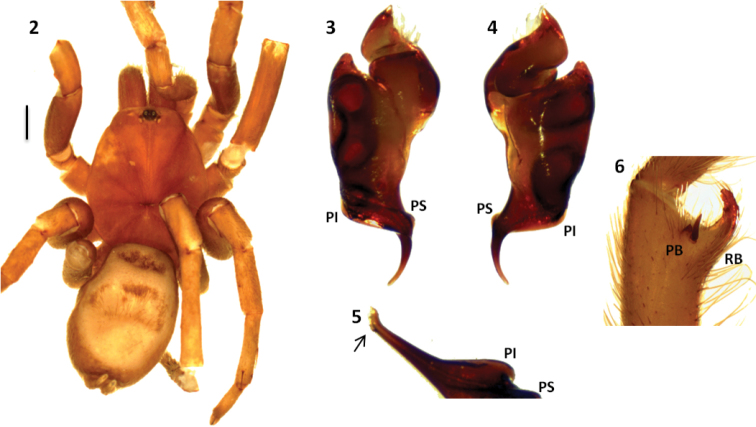
*Melloleitaoina crassifemur*. **2** male holotype, dorsal view **3–5** left palpal bulb, **3** prolateral view **4** retrolateral view **5** detail of apex widened **6** left tibial apophysis (subapical spine on retrolateral branch RB lost). Scale bar = 1 mm.

#### Re-description.

Holotype male (MACN-Ar 2285): total length, not including chelicerae or spinnerets, 14.1, carapace length 5.9, width 5.3. Color (in alcohol): cephalotorax, legs light brown, abdomen grayish brown. Anterior eye row slightly procurved, posterior recurved. Eyes and interdistances: AME 0.18, ALE 0.30, PME 0.14, AME-ALE 0.06, PME-PME 0.38, PME-PLE 0.04, ALE-PLE 0.08, AME-PME 0.04, ALE-ALE 0.40. OQ length 0.50, width 0.96, clypeus 0.14. Fovea transverse, procurved, width 0.70. Chelicerae with 10/12 well-developed teeth on furrow promargin, few small teeth on the proximal area of furrow. Labium length 0.68, width 1.07 with 7 cuspules. Maxillae with 74 cuspules. Sternum length 2.70, width 2.70. Tarsi I-IV scopula widely divided, by conical setae thicker and longer. Tarsal claws with 2-3 small teeth on proximal half, near the inner edge. Sparse scopulae on metatarsi; metatarsi I-III apically scopulate, IV without scopula. Tibia I with prolatero-ventral distal apophysis with two very unequal branches (Fig. 6); PB subtriangular, small, with basal curved spine, much longer than branch, RB curved, much larger than PB with internal medial spine that exceeds length of branch. Metatarsus I slightly curved, flexion on RB. Femur III very thickened. Type III-IV intermediate urticating setae present. PMS well-developed, PLS normal, apical segment digitiform. Palpal organ piriform with the embolus very curved, two prolateral keels (PI and PS) present, subequal, apex widened (Figs 3–5).

Spination. Femora: palp, I-IV 0. Patellae: palp, I-IV 0. Tibiae: palp 0, I 0, II 1P, III 2V, 1P, 2R, IV 1R. Metatarsi: I 1V, II 1V, III 3V, 2P, IV 4V, 2P, 1R. Tarsi: palp, I-IV 0.

Legs and palpal segments lengths (femur/patella/tibia/metatarsus/tarsus). Palp: 3.1/2.0/2.5/1.2 total 8.8; I: 5.5/3.5/4.4/3.8/2.4 total 19.6; II: 4.9/1.9/3.6/4.4/2.3 total 17.1; III: 3.5/2.0/2.8/3.7/2.2 total 14.2; IV: 6.0/2.2/5.0/6.3/2.5 total 22.

#### Remarks.

The female *Melloleitaoina crassifemur* was described thirteen years after the original description of the male holotype of the species. This female specimen was assigned as *Melloleitaoina crassifemur* because it was collected near the male type locality. We examined male specimens from the same locality of this female and found important morphological differences between these males and the holotype *Melloleitaoina crassifemur*, as the palpal bulb shape, the presence of a conspicuous triangular tooth on the embolus (Figs 16 and 17) and the spiniform setae on coxae III and IV (Figs 19–21), also it present on the female. Taking into account that these new males are sympatric with the female attributed to *Melloleitaoina crassifemur* we considered them as conspecific, and are here described as a new species.

### Melloleitaoina mutquina sp. n.

http://zoobank.org/ECCA9985-CFAE-4CBE-98EE-30154F1CD4E6

http://species-id.net/wiki/Melloleitaoina_mutquina

Figs 7–11

#### Material examined.

Known only from types.

#### Type material.

Holotype male from Argentina, Catamarca, Mutquín, 1500m above sea level, 28°19'0"S, 66°10'0"W, 2-ii-1981, E. Maury leg. (MACN-Ar 7737).

#### Diagnosis.

Male differs from other *Melloleitaoina* species by the palpal bulb morphology with the embolus less curved, absence of triangular tooth, PS very flat and apex widened (Figs 9 and 10). Females unknown.

**Figures 7–11.**
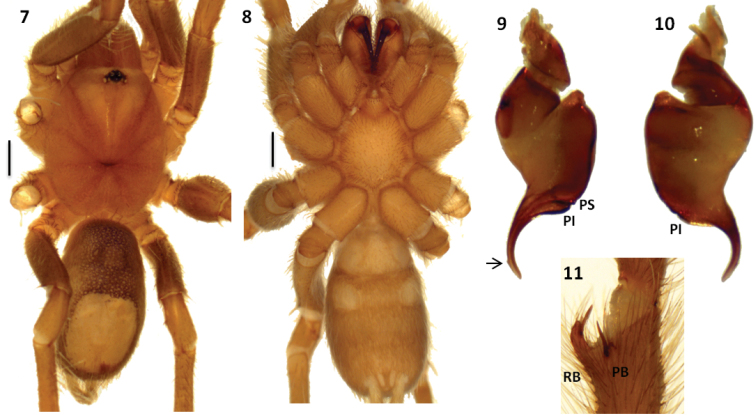
*Melloleitaoina mutquina*. **7–8** male holotype **7** dorsal view **8** ventral view **9–10** right palpal bulb **9** prolateral view **10** retrolateral view **11** right tibial apophysis. Arrow indicates apex widened. Scale bars = 1 mm.

#### Description.

Holotype male (MACN-Ar 7737): total length, not including chelicerae or spinnerets, 9.4, carapace length 4.1, width 3.7. Color (in alcohol): cephalotorax, legs light reddish brown, cephalotorax with few brown and golden setae, abdomen brown with a patch of urticating setae golden brown. Anterior eye row procurved, posterior slightly recurved. Eyes and interdistances: AME 0.15, ALE 0.20, PME 0.11, PLE 0.15, AME-AME 0.11, AME-ALE 0.066, PME-PME 0.33, PME-PLE 0.022, ALE-PLE 0.077, AME-PME 0.055, ALE-ALE 0.40. OQ length 0.68, width 0.61, clypeus 0.022. Fovea transverse, procurved, width 0.66. Chelicerae with 10/9 well-developed teeth on furrow promargin, 5/3 small teeth on the proximal area of furrow. Labium length 0.48, width 0.78, with 6 cuspules. Maxillae with 38/39 cuspules. Sternum length 1.9, width 1.9. Tarsi I-IV scopula widely divided. Tarsal claws with 1-4 small teeth on proximal half, ventral midline. Sparse scopulae on metatarsi; metatarsus I scopulate on distal third, II apically, III and IV without scopula. Tibia I with prolatero-ventral distal apophysis with two very unequal branches (Fig. 11); PB subtriangular, small, with basal curved spine, much longer than branch, RB curved, around ten times bigger than PB with internal medial spine that exceeds length of branch. Metatarsus I slightly curved, flexion on RB. Femur III thickened. Type III-IV intermediate urticating setae present. Palpal organ piriform with the embolus slightly curved, two prolateral keels (PI and PS) present, PS very flat, apex widened (Figs 9 and 10).

Spination. Femora: palp 0; I 0; II 0; III 0; IV 0. Patellae: palp 0; I 0; II 0; III 0; IV 0. Tibiae: palp 0; I 0; II 1P; III 1V, 1P; IV 1V, 1P, 1R. Metatarsi: I 1V; II 1V; III 3V, 2P; IV 4V, 1P. Tarsi: palp, I-IV 0.

Legs and palpal segments lengths (femur/patella/tibia/metatarsus/tarsus). Palp: 2.2/1.3/1.5/0.8 total 5.8; I: 3.5/2.0/2.9/2.1/1.4 total 11.9; II: 3.0/1.7/2.3/2.1/1.4 total 10.5; III: 2.7/1.4/1.8/2.3/1.4 total 9.6; IV: 3.8/1.7/3.0/4.0/1.8 total 14.3.

#### Etymology.

The specific epithet mutquina is a noun taken in apposition and means place or thing to smell in Quichua language and refers to the locality of Mutquín, where this species is distributed. It denotes the aroma of the flora of the region that emerges especially after rains, perfuming the village of aromatics herbs.

### Melloleitaoina uru sp. n.

http://zoobank.org/60708C57-F159-46CC-9EB3-3FFCEEA3FE7A

http://species-id.net/wiki/Melloleitaoina_uru

Figs 12–21

Melloleitaoina crassifemur : Gerschman and Schiapelli 1973: 87, Fig. 50 (in part, female only). Syn n.

#### Material examined.

**Type material.** Holotype male from Argentina, Salta, 37,5 Km O. Hickmann, 235m above sea level, 23°11'60"S, 63°34'0"W, Goloboff, Coyle, Bennet leg. (MACN-Ar 26042). Paratypes: female from Argentina, Salta, Aguaray, Punilla, 570m above sea level, 22°16'0"S, 63°43'60"W, iv-1948, Biraben leg. (MACN-Ar 6542); 2 males and 1 female, with the same data (MACN-Ar 6543); male from Argentina, Salta, Campamento Vespucio, 450m above sea level, 22°36'0"S, 63°49'0"W, 10-13-v-1988, Goloboff leg. (MACN-Ar 26043); 2 females from Argentina, Salta, Pocitos (Salvador Mazza), 800m above sea level, 22°4'0"S, 63°43'0"W, xi-1951; Biraben leg. (MACN-Ar 6544).

**Other material.** Juvenile from Argentina, Salta, Aguaray, Punilla, 22°16'0"S, 63°43'60"W, iv-1948, Biraben leg. (MACN-Ar 6542); 3 juveniles from Argentina, Salta, Pocitos (Salvador Mazza), 22°4'0"S, 63°43'0"W, xi-1951; Biraben leg. (MACN-Ar 6544).

#### Diagnosis.

Males differ from other *Melloleitoina* species by the palpal bulb morphology with very curved embolus with a conspicuous subapical triangular tooth and well-developed PI and PS (Figs 16–18). Females differ from other *Melloleitaoina* species by the shape of the spermathecae with elongated seminal receptacles with small granules (Fig. 15).

**Figures 12–21.**
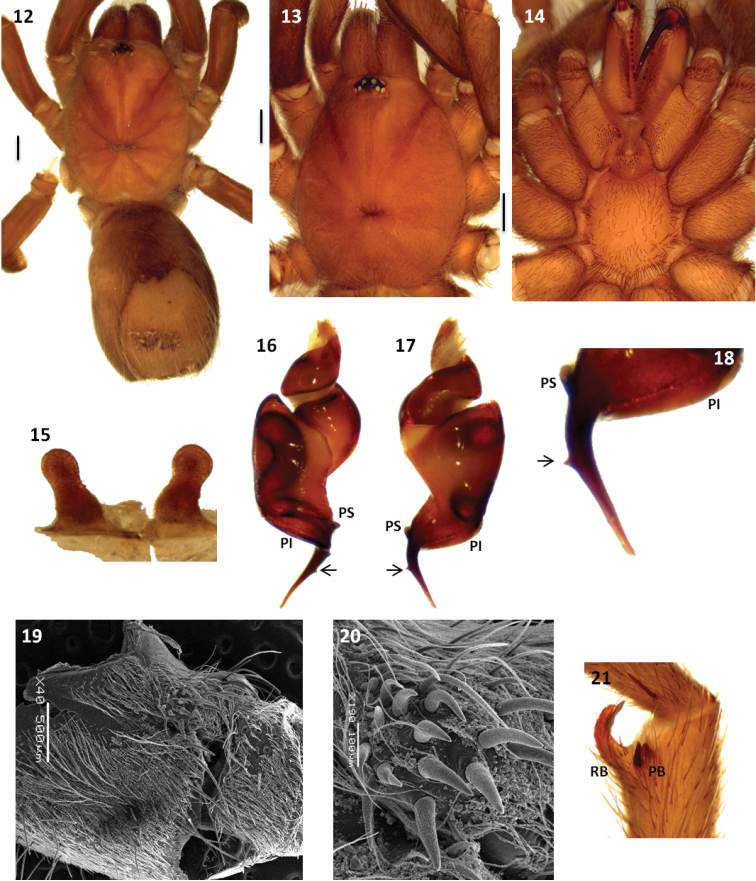
*Melloleitaoina uru*. **12** female, dorsal view **13–14** male holotype **13** cephalotorax **14** sternum, labium, maxillae and quelicerae **15** spermathecae **16–18** left palpal bulb **16** prolateral view **17** retrolateral view **18** detail of triangular tooth on embolus **19–20** coxa III **19** prolateral view **20** detail of spiniform setae **21** right tibial apophysis. Arrow indicates triangular tooth on embolus. Scale bars black = 1 mm.

#### Description.

Holotype male (MACN-Ar 26042): total length, not including chelicerae or spinnerets, 13.8, carapace length 6.5, width 5.5. Color (in alcohol): cephalotorax, legs clear reddish brown, cephalotorax with few brown and golden setae, abdomen brown with a patch urticating setae golden brown. Anterior eye row slightly procurved, posterior slightly recurved. Eyes and interdistances: AME 0.17, ALE 0.27, PME 0.17, PLE 0.22, AME-AME 0.12, AME-ALE 0.06, PME-PME 0.43, PME-PLE 0.025, ALE-PLE 0.06, AME-PME 0.075, ALE-ALE 0.50. OQ length 0.97, width 0.72, clypeus 0.025. Fovea transverse, procurved, width 0.75. Chelicerae with 8 well-developed teeth on furrow promargin, 12/14 small teeth on the proximal area of furrow. Labium length 0.57, width 1.45, with 11 cuspules. Maxillae with 109/114 cuspules. Sternum length 2.6, width 2.6. Coxae III and IV with spiniform setae on promargin (Figs 19–20). Tarsi I-IV scopula widely divided. Tarsal claws with 3 small teeth on proximal half, ventral midline. Sparse scopulae on metatarsi; metatarsus I scopulate on distal half, II on distal third, III apically, IV without scopula. Tibia I with prolatero-ventral distal apophysis with two very unequal branches (Fig. 21); PB subtriangular, small, with basal spine, similar size to the branch, RB curved, at least five times bigger than PB with internal medial spine that exceeds length of branch. Metatarsus I slightly curved, flexion on RB. Femur III thickened. Type III-IV intermediate urticating setae present. Palpal organ piriform with the embolus very curved and with a conspicuous subapical triangular tooth, two prolateral keels (PI and PS) present, subequal (Figs 16–18).

Spination. Femora: palp 1P; I 1P; II 1P; III 1P, 1R; IV 1R. Patellae: palp 0; I 0; II 0; III 1P; IV 0. Tibiae: palp 2P; I 2V, 1P; II 3V, 1P; III 7V, 2P, 2R; IV 7V, 2P, 3R. Metatarsi: I 2V, 1P; II 2V, 1P; III 9V, 3P, 2R; IV 10V, 3P, 3R. Tarsi: palp, I-IV 0.

Legs and palpal segments lengths (femur/patella/tibia/metatarsus/tarsus). Palp: 3.2/2.0/2.7/1.1 total 9.0; I: 5.3/2.7/4.0/3.6/2.3 total 17.9; II: 4.7/2.4/3.3/3.2/2.1 total 15.7; III: 3.9/1.9/2.5/3.7/2.1 total 14.1; IV: 5.9/2.4/5.0/6.6/2.3 total 22.2.

Paratype female (MACN-Ar 6542): total length, not including chelicerae or spinnerets, 14.8, carapace length 6.6, width 5.7. Color (in alcohol): as in male. Anterior eye row slightly procurved, posterior slightly recurved. Eyes and interdistances: AME 0.17, ALE 0.31, PME 0.17, PLE 0.25, AME-AME 0.15, AME-ALE 0.06, PME-PME 0.4, PME-PLE 0.025, ALE-PLE 0.075, AME-PME 0.10, ALE-ALE 0.56. OQ length 1.02, width 0.95, clypeus 0.025. Fovea transverse, procurved, width 1.12. Chelicerae with 10 well-developed teeth on furrow promargin, 12 small teeth on the proximal area of furrow. Labium length 0.62, width 1.42, with 6 cuspules. Maxillae with 96/89 cuspules. Sternum length 2.9, width 2.9. Coxae III and IV with spiniform setae on promargin (Figs 19–20). Tarsi palp, I-IV scopula widely divided. Tarsal claws with 3 small teeth on proximal half, ventral midline. Sparse scopulae on metatarsi; metatarsus I scopulate on distal half, II on distal third, III and IV without scopula. Type IV urticating setae present. Spermathecae with two elongated seminal receptacles with small granules (Fig. 15).

Spination. Femora: palp 0; I 0; II 1P; III 1P, 1R; IV 0. Patellae: palp 0; I 0; II 0; III 1P; IV 0. Tibiae: palp 3V; I 0; II 0; III 4V, 3P, 1R; IV 7V, 3P, 2R. Metatarsi: I 2V; II 3V; III 6V, 3P, 2R; IV 10V, 3P, 3R. Tarsi: palp, I-IV 0.

Legs and palpal segments lengths (femur/patella/tibia/metatarsus/tarsus). Palp: 3.2/2.4/2.0/2.1 total 9.7; I: 4.0/2.5/3.0/2.4/1.5 total 13.4; II: 3.8/2.5/2.7/2.4/1.6 total 13.0; III: 3.2/2.4/2.0/3.0/1.6 total 12.2; IV: 4.7/2.6/4.0/5.0/2.0 total 18.3.

#### Variation.

Males and females total length 9.2-16.5. Labium cuspules 6-14.

#### Etymology.

The specific epithet is a noun taken in apposition and refers to an ancient legend Quichua, from the northern limit of Argentina, about the princess Inca Uru, who by their whims and bad government was transformed by the gods into a spider and forced to endlessly work weaving.

### Melloleitaoina yupanqui sp. n.

http://zoobank.org/3BD376C4-CA74-4A6A-8D2F-99445C54587E

http://species-id.net/wiki/Melloleitaoina_yupanqui

Figs 22–29

#### Material examined.

Known only from types.

#### Type material.

Holotype male from Argentina, Jujuy, P. Nacional Calilegua, Seccional Aguas Negras, 605m above sea level (GPS), 23°45'43.3"S, 64°51'04.7"W (± 10m, WGS84), 06-11-xii-2008, C. Grismado, M. Izquierdo, F. Labarque, G. Rubio, M. Burger, P. Michalik, P. Carrera, A. Ojanguren and C. Mattoni leg. (MAC-Ar 26041). Paratype female, same data as the holotype (MAC-Ar 26044).

#### Diagnosis.

Male differs from other *Melloleitaoina* species by the palpal bulb morphology with a discontinuous PS, formed by two separate keels, very curved embolus without triangular tooth, well-developed PI and PS, and apex widened (Figs 26 and 28). Female differs from other *Melloleitaoina* species by the shape of the spermathecae with short seminal receptacles with large granules (Fig. 25).

**Figures 22–29.**
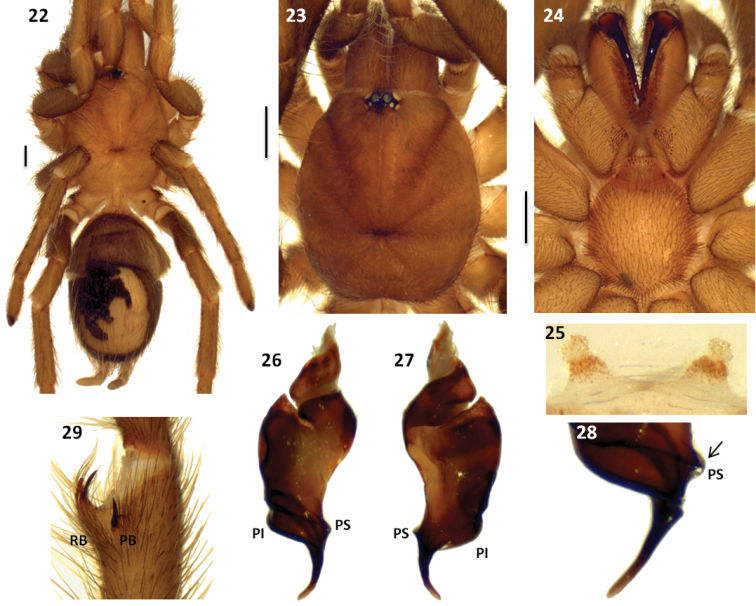
*Melloleitaoina yupanqui*. **22** female, dorsal view **23–24** male holotype **23** cephalothorax **24** sternum, labium, maxillae and quelicerae **25** spermathecae **26–28** left palpal bulb **26** prolateral view **27** retrolateral view **28** detail of embolus showing PS discontinuous **29** right tibial apophysis. Scale bars = 1 mm.

#### Description.

Holotype male (MAC-Ar 26041): total length, not including chelicerae or spinnerets, 9.3, carapace length 4.3, width 4.0. Color (in alcohol): cephalotorax, legs light reddish brown, cephalotorax with few brown and golden setae, legs darker, mainly femora, abdomen brown with a patch of urticating setae golden brown. Anterior eye row slightly procurved, posterior slightly recurved. Eyes and interdistances: AME 0.17, ALE 0.22, PME 0.12, PLE 0.16, AME-AME 0.088, AME-ALE 0.033, PME-PME 0.36, PME-PLE 0.022, ALE-PLE 0.055, AME-PME 0.022, ALE-ALE 0.39. OQ length 0.75, width 0.55, clypeus 0.022. Fovea transverse, procurved, width 0.68. Chelicerae with 11 well-developed teeth on furrow promargin, 8/10 small teeth on the proximal area of furrow. Labium length 0.48, width 0.88, with 8 cuspules. Maxillae with 53/51 cuspules. Sternum length 2.0, width 2.0. Tarsi I-IV scopula widely divided. Tarsal claws with 2 small teeth on proximal half, near the inner edge. Sparse scopulae on metatarsi; metatarsus I scopulate on distal half, II scopulate on distal third, III only apically scopulate, IV without scopula. Tibia I with prolatero-ventral distal apophysis with two very unequal branches (Fig. 29); PB very short with basal spine, much longer than branch, RB curved, around ten times bigger than PB with internal medial spine that exceeds the length of branch. Metatarsus I slightly curved, flexion on RB. Femur III thickened. Type III-IV intermediate urticating setae present. Palpal organ piriform with the embolus very curved, two prolateral keels (PI and PS) present, discontinuous PS, formed by two keels, apex widened (Figs 26–28).

Spination. Femora: 0. Patellae: 0. Tibiae: palp 0; I 1P; II 0; III 2V, 2P, 1R; IV 4V, 2R. Metatarsi: I 1V; II 1V; III 6V, 3P, 1R; IV 8V, 1P, 2R. Tarsi: palp, I-IV 0.

Legs and palpal segments lengths (femur/patella/tibia/metatarsus/tarsus). Palp: 2.4/1.3/1.9/0.7 total 6.3; I: 3.9/2.2/3.3/2.7/1.7 total 13.8; II: 3.6/2.0/2.6/2.4/1.7 total 12.3; III: 2.9/1.4/1.8/2.5/1.6 total 10.2; IV: 4.6/1.7/3.6/4.9/2.2 total 17.0.

Paratype female (MAC-Ar 26044): total length, not including chelicerae or spinnerets, 10.6, carapace length 4.9, width 4.0. Color (in alcohol): as in male, but lighter. Anterior eye row slightly procurved, posterior slightly recurved. Eyes and interdistances: AME 0.17, ALE 0.31, PME 0.19, PLE 0.21, AME-AME 0.08, AME-ALE 0.04, PME-PME 0.31, PME-PLE 0.022, ALE-PLE 0.044, AME-PME 0.066, ALE-ALE 0.39. OQ length 0.81, width 0.66, clypeus 0.022. Fovea transverse, procurved, width 0.71. Chelicerae with 9 well-developed teeth on furrow promargin, 15/14 small teeth on the proximal area of furrow. Labium length 0.55, width 1.1, with 8 cuspules. Maxillae with 90/87 cuspules. Sternum length 2.1, width 2.1. Coxae III and IV with spiniform setae on promargin (as Figs 19 and 20). Tarsi palp, I-IV scopula widely divided. Tarsal claws with 2 small teeth on proximal half, near the inner edge. Sparse scopulae on metatarsi; metatarsus I scopulate on distal half, II on distal third, III and IV without scopula. Type IV urticating setae present. Spermathecae with two short seminal receptacles with large granules (Fig. 25).

Spination. Femora: palp 1P; I 1P; II 1P; III 1P, 1R; IV 0. Patellae: palp 0; I 0; II 0; III 1P; IV 0. Tibiae: palp 4V; I0; II 0; III 5V, 2P, 2R; IV 5V, 2R. Metatarsi: I 2V; II 2V; III 9V, 3P, 2R; IV 7V, 2P, 2R. Tarsi: palp, I-IV 0.

Legs and palpal segments lengths (femur/patella/tibia/metatarsus/tarsus). Palp: 2.5/1.6/1.7/1.6 total 7.4; I: 3.3/2.2/2.5/1.8/1.4 total length 11.2; II: 2.9/1.9/2.0/1.7/1.4 total 9.9; III: 2.6/1.5/1.7/2.3/1.4 total 9.5; IV: 3.7/1.7/2.8/3.3/1.7 total 13.2.

#### Etymology.

The specific epithet is a patronym in honor to the most important Argentine musician of folklore Atahualpa Yupanqui, pseudonym of Héctor Roberto Chavero Aramburu (Juan A. de la Peña, Argentina, 1908 – Nimes, Francia, 1992).

### Key to males of *Melloleitaoina* species

**Table:** 

1	Palpal bulb with a triangular tooth on the embolus (Figs 16–18)	*Melloleitaoina uru*
–	Palpal bulb without a triangular tooth on the embolus	2
2	Embolus slightly curved and very flat PS (Figs 9 and 10)	*Melloleitaoina mutquina*
–	Embolus very curved and well developed PS	3
3	Embolus with continues PS (Figs 3 and 4)	*Melloleitaoina crassifemur*
	Embolus with discontinous PS (Figs 26–28)	*Melloleitaoina yupanqui*

## Supplementary Material

XML Treatment for Melloleitaoina

XML Treatment for Melloleitaoina crassifemur

XML Treatment for Melloleitaoina mutquina

XML Treatment for Melloleitaoina uru

XML Treatment for Melloleitaoina yupanqui
